# Gerald Hubert Leatherman DSc FDS FFD DOdont (1903–1991), the World Dental Federation, dental hygienists and the promotion of oral health

**DOI:** 10.1177/09677720211000354

**Published:** 2021-08-03

**Authors:** Stanley Gelbier

**Affiliations:** Unit for the History of Dentistry, Faculty of Dentistry, Oral & Craniofacial Science, King’s College London, Guy’s Hospital, London, UK

**Keywords:** World Dental Federation (Federation Dentaire International), Gerald Leatherman, first UK dental hygienist training programme

## Abstract

In 1994 a ‘Dr Gerald Leatherman Award' was established by the British Dental Hygienists' Association to honour Leatherman. But who was he? And why was he associated with this named award? There are many facets to the Leatherman story: the first training of UK dental hygienists, support for their association, promotion of oral health in many ways and, perhaps especially, his work for the World Dental Federation (FDI).

## Gerald H Leatherman

Gerald (Gerry) was born in in west London on 18 February 1903. At the age of seven he and his mother travelled to South Africa. There he continued his education at King Edward VII School in Johannesburg. He then studied chemistry and physics at Witwatersrand University.

In 1918 the young Gerald visited the dental practice of his father's friend, Lemuel Morgan-Davis. He was fascinated by what he saw, including crowns, dentures and the use of plaster of Paris for impressions.^1^ Morgan-Davis arranged for Gerald to later apply to his *alma mater,* Harvard Dental School in Boston. He worked his way to pay for the 31 day voyage from Durban to New York City, arriving on the small freighter on 15 September 1920.

Gerald enrolled at Harvard at the very early age of 17 years. During his four years there Gerald worked during the four-months summer holidays at a hotel and a steel furniture factory and also travelled. He said: “I learned that dentists – addressed as doctors – were respected, and even dental students could be proud of their education, training and future profession.” Gerald Leatherman graduated with a Doctorate in Dental Surgery from Harvard University Dental School *cum laude* in 1924.

He then enrolled at Guy's Hospital Dental School in London. By December 1924 he had satisfied all the requirements and passed the examinations for the Licence in Dental Surgery of the Royal College of Surgeons of England.

He went back to South Africa where he intended to practise. However, on finding that Lemuel Morgan-Davis had gone to America, Leatherman returned to London, arriving on 8 March 1925. He then registered his LDS – but not the DDS – with the Dental Board of the UK.

By 1926 Leatherman started a private practice at 11 Devonshire Place, off Devonshire Street, W1. Based on oral hygiene and prevention combined with high quality restorative dentistry, he had a very successful practice. Leatherman was forever seeking better ways to provide care. He once travelled to Stockholm to see the new local anaesthetic Xylocaine being used. He brought it back to London to use on his patients.^2^

In 1931, the year he went to Paris for an FDI meeting, he was registered at 17 Harley Street. By 1942 he was listed at 35 Devonshire Place (although serving in the RAF).

## Leatherman and the World Dental Federation (FDI)

**Figure 1.**
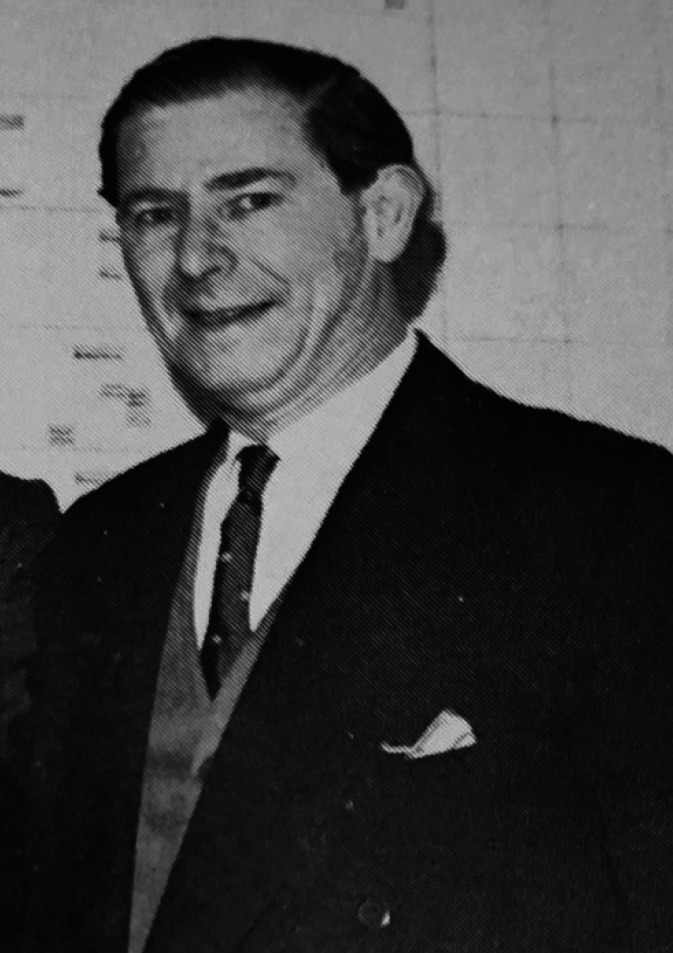
Gerald Leatherman.

Not surprisingly Dame Margaret Seward described Leatherman (Figure 1) as ‘The Father of World Dentistry’. However his first contact with the Fédération Dentaire Internationale (FDI) was minimal; at the 1931 World Dental Congress in Paris, the city where it started in 1900. However, according to its history, he showed little interest in the FDI at that time. After visiting the Exhibition he went off to play golf.^3^ Yet by 1947 he was assistant secretary of the FDI.

The association was badly hit by World War II. In 1947 a small FDI Congress in Boston was attached to the 88th conference of the American Dental Association. Marie Ferdinand Watry of Belgium was elected executive secretary with Leatherman and J de Wever of Belgium as assistant secretaries.

President Charles Nord of Norway and secretary Watry said there was a need for change, with ‘new men’. The old guard were a handful of men who ran the FDI with their own money (“socially useful way of seeing the capitals of Europe and making foreign friends”).^4^ The new world needed a proper financial footing. Oren A Oliver ran a massively successful recruitment drive in the USA, the income from which almost single-handedly balanced the books. Leatherman ran a similar but smaller drive in the UK. He also negotiated a contract with Messrs Cassell to produce an International Dental Journal.

What really brought Leatherman to people’s notice was his work on two committees. In 1950 Leatherman became secretary of a committee of reorganisation under the chairmanship of **Sir Wilfred** Fish. Their suggestions were “about to drag the FDI kicking and screaming … into the second half of the 20th century”.^5^

Under Fish’s presidency Leatherman chaired the organising committee for the 1,95,211th International Congress to be held in London. It was highly successful, with 3,940 people from 67 countries, held at the Royal Festival Hall. Its daily 4-page newspaper, the *Congress Courier* reported its news. For the first time there was simultaneous translation of sessions into five languages. The science programme was opened by Fish’s friend Sir Alexander Fleming. 3,500 people attended a concert by the London Philharmonic Orchestra. It was the first organisation to demonstrate that the Festival Hall’s facilities were excellent for conferences.

As the Congress closed the FDI’s General Assembly met on 26 July and elected a new council. Alfred Ernest Rowlett remained as president of honour with Oren Oliver of the USA as president. Leatherman became the secretary general, a post he held for 28 years. Another British dentist, W Stewart Ross, became chair of the council, a new position. Many people saw the London Congress as the beginning of the modern FDI.^6^ Much was due to the “energetic Leatherman”, Rowlett and Harold Hillenbrand, Executive Director of the American Dental Association.

Leatherman was Rowlett’s protégé. The latter had groomed Leatherman for some time and urged him to take a leading role. Hillenbrand recognised in Leatherman a man as tough-minded as himself, so backed him and gave much advice. He said: “Gerry had the courage and the essential nastiness, to get the job done.”^7^

When Leatherman became secretary Hillenbrand was vice-president, a position from which he could offer much support, as did Oliver the new president who had single-handedly raised the cash balances. Together they changed the FDI from gatherings of prominent individuals interested in international dentistry to one of meetings of delegates from national associations – a world parliament.

Leatherman ran the organisation out of 35 Devonshire Place with four staff, with a further two in the Hague to support the treasurer, J Stork.

In 1951 Leatherman issued a stencilled newsletter: 1,000 copies in English and French. By 1960 there were 6,000 in English, French, Spanish, German and Italian. The news section in their journal was no longer needed.

Part of Leatherman’s early efforts was to secure links with WHO, a relationship much driven by Stork. Over the years that link became very strong.

The title of executive secretary was replaced in 1970, when Leatherman was elected executive director of the FDI. He continued to serve until 1975 at which time he retired after serving in that leadership position for over 20 years. By then the FDI had 73 full member associations and 10,000 individual members. At the close of the 1975 meeting he was elected as executive director-emeritus.

Dame Margaret Seward regarded Leatherman as her mentor. In 1981 she was due to attend a World Dental Congress in Rio de Janeiro, Brazil. For many reasons, including worries about lack of safety, lots of people decided not to go and it was cancelled. Seward recalled Leatherman saying it would be good for her to attend an annual meeting of the American Dental Association so went instead to Kansas City.^8^ Good to his word Leatherman introduced her to ‘movers and shakers’ of American dentistry and many doyens of the world dento-political arena, and took her to some committee meetings. Not surprisingly she later became editor of th IDJ, sucked in by Leatherman.

## Leatherman and dental hygienists

During his LDS viva exam at Guy’s Hospital Sir William Kelsey Fry asked Leatherman what was the most important thing he learned in America. His immediate answer was “the value of good oral hygiene”.^9^ Whilst in the USA Leatherman experienced a new group of auxiliary personnel, dental hygienists, as well as oral health clinics. He realised their potential and brought his ideas back to the UK.

In 1907, a Connecticut dentist, Alfred Fones, knew the need for good mouth care to reduce oral bacteria which cause caries. He therefore trained his cousin, Irene Newman, to perform dental prophylaxis on his patients. This led Fones to establish America’s first dental hygiene programme in 1913, in Bridgeport, Connecticut. The first graduates were employed in school-based settings to emphasize the importance of oral hygiene to children. In 1917 Newman became the first licensed dental hygienist. By 1952, all fifty states had licensed hygienists.

Leatherman began influencing similarly-minded colleagues which led eventually to recognition of dental hygienists as an important member of the dental team. But there was a long way to go.

Leatherman played a major role in promoting the use of dental hygienists in UK preventive care. He devoted many years to dental health promotion and raising the profile of hygienists. Leatherman was actively involved with the British Dental Hygienists Association. His role in the first UK hygienist training school should be remembered with gratitude.

## The first RAF dental hygienists

Early in World War II the Royal Air Force found a serious oral health problem amongst all ranks, including flight crews. Acute necrotising gingivitis and dental sepsis were major issues. They led to the loss of many operational flying hours and lost engineering and administrative support.

With insufficient dental officers it was essential to do something quickly. so they rapidly had to think of something else to ensure maximum efficiency and the avoidance of wasted of man-hours.

In 1942 Sir William Kelsey-Fry, a civilian consultant to the RAF, suggested to the Director of Dental Services that dental hygienists could help to alleviate the problems in the neglected mouths of young recruits with little basic oral hygiene knowledge. He said much gross dental disease could be prevented through education.

As a result a trial scheme to train hygienists was established at the RAF Medical Training Establishment at Sidmouth, Devon based on Fones’ scheme.^10^ Specially selected WAAFs^11^ who were dental clerk/orderlies (dental nurses) underwent sixteen weeks training after which they could scale and polish teeth and educate patients on prevention.

The people largely responsible for implementing the programme were Squadron Leader James Smith and a young RAF volunteer reserve dentist, Flight Lieutenant Gerald Leatherman. The latter brought to the scheme knowledge he had gained in the USA, which Kelsey-Fry might have remebered. The UK Dental Board reminded the RAF that hygienists were not a legal entity but the Force responded that there was a war on and that ‘needs must’. After the war Leatherman led the move of the hygienist training school to the RAF Dental Training Establishment in Halton, Buckinghamshire. By then the course lasted nine months.

These activities taught Leatherman much about dental politics, discovering how bitterly against hygienists were many members of the profession.

After the war there was increasing interest in hygienists, especially from school dental services as they had manpower shortages. Of course some dentists had experienced their benefits in the RAF. The Ministry of Health became interested and the government sponsored a trial training scheme at the Eastman Dental Hospital from 1949. However it closed in 1954.^12^

In 1944 Cyril Bowdler Henry had confirmed in the British Medical Journal the importance of dental hygienists and described how he was part of a 1928 experiment that would see hygienists only working in civil (charitable) practices.^13^ Rather surprisingly he told how for many years he employed a female dental surgeon just to treat gum disease. He went on to credit the RAF for taking forward this cost-effective step.

## Dental hygienists and the ‘Dr Gerald Leatherman Award'

As more hygienists were trained, albeit in small number, they felt a need to band together. The British Dental Hygienists' Association was formed in July 1949 at the Eastman Dental Clinic. Vera Creaton was elected chairman, with Ann Hamer as secretary.

Right from the beginning and for many years Leatherman gave them enormous support. In return he was the president of the BDHA from 1949 to 1957 and then honorary president until he died in 1991.

The Dentists Act 1957 led to the new General Dental Council becoming the regulating body for hygienists through the Central Examination Body for Dental Hygienists, which awarded, set and monitored standards for dental hygienists training and examinations. The Act also led to the Ancillary Dental Workers Regulations 1957. They laid down for the first time in law the role and responsibilities of hygienists.

The Council set up a Roll of Dental Hygienists on which all practising hygienists must have their names entered. Until 1961 hygienists who undertook service examinations were permitted to register them with the GDC. It aligned them with those who trained at the small number of dental hospitals then providing dental hygienist training funded by the Ministry of Health.

In May 1958 the GDC issued its first recommendations on courses of instruction for hygienists.^14^ They were initially trained for a period of no less than nine months and qualified with a Certificate of Proficiency in Oral Hygiene. This entitled them to register with the GDC and practice as a hygienist in accordance with the Dentists Act. In so doing it protected title Dental Hygienist in law. A nother step forward came in 1970 when the GDC approved the use of the letters EDH for enrolled hygienists.

In order to recognise the work and support of Leatherman, the ‘Dr Gerald Leatherman Award' was established in 1994 to perpetuate and honour the name of this great man. It is held in the highest regard by hygienists. The award is the only one nominated and agreed upon by members. It reflects true dedication, professionalism and determination for the greater good of all the profession. Winners have included people who worked tirelessly behind the scenes as well as those who laid the foundations for the society. This prestigious award can be given to any individual, who has shown consistent support to the profession or the association.

The Award takes the form of a lapel pin bearing the words ‘Dr Leatherman Award' and a certificate to commemorate the date that the presentation was made. The first and most recent recipients were Jean Bailey (1994) and Christina Chatfield (2020).

## Board of the Faculty of Dental Surgery

Leatherman also brought his knowledge and skills to the Royal College of Surgeons of England. He served on its Faculty of Dental Surgery board from 1952 to 1966. He was always on the lookout for dentists worth encouraging. Dame Margaret Seward said^12^ Leatherman was her mentor. When she was editor of the British Dental Journal he encouraged her to pursue other interests. At the time he was a staunch advocate of the work of the board. It had a dean, vice-dean and 16 others: “All Caucasian males in 1974.” Leatherman encouraged Margaret to seek election. She wondered what she, a female school dentist with two children, could offer to obtain one of the four seats up for election. Most of the other candidates were consultant types. Although not elected Margaret came top of the near misses. By 1980 she was editor of the BDJ, a member of the GDC and very well known. She was elected out of a field of twenty. She was grateful to Leatherman for giving her a push.

## The British Dental Association

Leatherman was president of the Metropolitan Branch of the BDA in 1954. In 1975 the association afforded him honorary membership of the association.

## British Society of Periodontology

In 1948 Gerald Leatherman, George Cross,^15^ Sam Cripps and Cyril de Vere Green discussed a possible society for people interested in gum and bone diease. It went ahead as the British Society of Periodontology, with Sir Wilfred Fish as the president.

## Personal life

Leatherman had two daughters with his first wife Constance who he divorced in 1950. His second marriage to Margaret (Peggie) “brought him much joy and satisfaction”.^16^ She died tragically in 1978.

He retired from practice aged 70, having continued to work part-time in all the years of his FDI administrative leadership. He left the FDI two years later.

Gerald Leatherman studied racehorse form and enjoyed frequent bets on them. An obituary points out that on the day he died from cancer on 11 December 1991 all racing was stopped – but because of frost rather than his death.^17^

## Honours and awards

During his lifetime Leatherman was recognized throughout the world for his contributions to the dental profession. He was elected to honorary FDSRCS Eng & Edin, FFD Ireland, fellow of The American College of Dentists, the Intemational College of Dentists, the Royal Australasian College of Dental Surgeons, The Royal College of Dentists of Canada, The Harriet Newell Research Society of Harvard University School of Dental Medicine, the Royal Society of Medicine (Odontological Section) and the Royal Society of Health.

He was awarded honorary doctorate degrees from Temple University School of Dentistry, Philadelphia and the University of Turkey. He was elected to over 37 honorary memberships throughout the world including the American Dental Association, the Australian Dental Association, the American Dental Society of Europe, the British Dental Association and the Canadian Dental Association. He published countless papers and gave innumerable lectures throughout the world during his 50 plus years of service to dentistry.

The Pierre Fauchard Academy recognised the late Dr Gerald H Leatherman as the 14th recipient into its International Hall of Fame of Dentistry on 29 June 2001.

The New York State Dental Association bestowed its Jarvie-Burkhart Award in 1979. The Jarvie-Burkhart Award, established in 1905, is named after former New York State Dental Association leaders and dental pioneers, William Jarvie and Harvey Burkhart.

Clearly Gerald Leatherman was someone who enjoyed all aspects of his life. He ran a very successful private practice for nearly fifty years. Leatherman’s involvement in dental politics was extensive and his peers worldwide recognized him.
